# Macrolide-Resistant *Mycoplasma pneumoniae* in Children, Ohio, USA

**DOI:** 10.3201/eid3112.251008

**Published:** 2025-12

**Authors:** Huanyu Wang, Tori Embry, Kathy Everhart, Morgan Szekely, Jeanette Taveras, Sophonie J. Oyeniran, Amy L. Leber

**Affiliations:** Nationwide Children’s Hospital, Columbus, Ohio, USA (H. Wang, T. Embry, K. Everhart, M. Szekely, J. Taveras, S.J. Oyeniran, A.L. Leber); The Ohio State University, Columbus (H. Wang, J. Taveras, S.J. Oyeniran, A.L. Leber)

**Keywords:** Mycoplasma pneumoniae, macrolide resistance, antimicrobial resistance, bacteria, respiratory infections, pediatrics, Ohio, United States

## Abstract

As the COVID-19 pandemic waned, *Mycoplasma pneumoniae* reemerged in the pediatric population in Ohio, USA. The rate of macrolide-resistant *M. pneumoniae* fluctuated by month, ranging from 0 to 8.7%, and mirrored the azithromycin prescribing rate. Real-time surveillance for macrolide-resistant *M. pneumoniae* provides accurate information for management of children with these infections.

*Mycoplasma pneumoniae* is a major pathogen associated with community-acquired respiratory infections in school-age children. Cyclic epidemics occur every few years (*1*). As the COVID-19 pandemic waned, *M. pneumoniae* reemerged in a patient population in Ohio, USA, in September 2023. Activity peaked in summer 2024. This elevated activity persisted through winter 2024–25 and declined in the spring 2025, resulting in the largest *M. pneumoniae* outbreak in central Ohio in a decade. 

Macrolides are the first-line treatment for *M. pneumoniae* infections (*2*). We previously reported an average of 2.4% macrolide-resistant *M. pneumoniae* (MRMp) rate during September 2023–September 2024, which showed resistance increasing over time (*3*). Here, we describe an overview of the entire outbreak and changes in MRMp rates during that period.

During September 1, 2023–May 27, 2025, we identified patients <21 years of age who had positive *M. pneumoniae* tests by multiplex respiratory molecular panel (RP2.1; BioFire Diagnostics, https://www.biofiredx.com) or laboratory-developed PCR (MPN-PCR). We tested a subset of remnant specimens for macrolide resistance (*3*). We extracted demographic information, laboratory findings, testing location, and hospitalization status from electronic medical charts. We obtained azithromycin prescription data from records of patients seen at Nationwide Children’s Hospital urgent care centers. We conducted data analyses by using GraphPad Software (https://www.graphpad.com). 

During the study period, 45,320 *M. pneumoniae* tests were performed. We identified 7,969 (17.6%) positive results from 7,649 unique patients during 7,740 medical encounters (Table; Appendix Figure). Positive test percentages began increasing in June 2024, peaked at 31% in July, and remained >20% until January 2025. The median age of patients with positive tests was 8.9 years (interquartile range [IQR] 5.8–12.0 years); 4,037 (52.8%) were male and 3,612 (47.2%) female. A total of 657 (8.5%) patients were hospitalized; 153 (2.0%) required intensive care unit (ICU) admission. Among the 795 patients with positive *M. pneumoniae* tests who had RP2.1 testing, 340 (42.8%) had co-detection of other respiratory viruses on the panel. We conducted an age-stratified analysis of resistance rates, testing location, hospitalization, ICU admission, and co-detection (Appendix Table 1).

**Table:** *Mycoplasma pneumoniae* testing volume, number of positive tests, sequencing volume, resistance detection, and azithromycin prescriptions, by month, Ohio, USA, September 2023–May 2025*

Month	Total cohort					Urgent care center cohort				
	No. samples tested	No. (%) positive	No. (%) sequenced	No. (%) resistant		No. samples tested	No. (%) positive	No. (%) sequenced	No. (%) resistant	Azithromycin prescription rate†	
2023											
Sep	818	5 (0.6)	0	NA		38	0	0	NA	4	
Oct	1,001	29 (2.9)	0	NA		55	6 (10.9)	0	NA	6	
Nov	1,348	59 (4.4)	0	NA		102	7 (6.9)	0	NA	8	
Dec	1,697	62(3.7)	0	NA		160	13 (8.1)	0	NA	9	
2024											
Jan	1,388	57 (4.1)	6 (10.5)	0		79	13 (16.5)	5 (38.5)	0	6	
Feb	1,303	29 (2.3)	6 (20.7)	0		76	4 (5.3)	2 (50.0)	0	5	
Mar	1,085	45 (4.2)	1 (2.2)	0		62	12 (19.4)	1 (8.3)	0	5	
Apr	965	56 (5.8)	27 (48.2)	0		73	14 (19.2)	10 (71.4)	0	6	
May	1,045	106 (10.1)	47 (44.3)	0		130	36 (27.7)	21 (58.3)	0	8	
Jun	1,020	266 (28.1)	145 (54.1)	1 (0.7)		251	126 (50.2)	82 (65.1)	1 (1.2)	25	
Jul	1,318	407 (30.9)	226 (55.5)	2 (0.9)		396	194 (48.7)	124 (64.3)	2 (1.6)	35	
Aug	2,009	642 (32.0)	285 (44.4)	10 (3.9)		761	343 (45.1)	180 (52.5)	4 (2.2)	51	
Sep	3,067	860 (28.0)	252 (29.3)	11 (4.4)		1,253	479 (38.2)	133 (27.8)	3 (3.0)	69	
Oct	5,657	1,831 (32.4)	299 (16.3)	26 (8.7)		2,084	826 (39.6)	108 (13.1)	6 (5.6)	109	
Nov	6,943	1,649 (23.8)	276 (16.7)	13 (4.7)		2,322	693 (29.8)	117 (16.9)	3 (2.6)	131	
Dec	6,210	1,288 (20.7)	270 (21.0)	17 (6.3)		1,976	572 (29.0)	120 (21.0)	6 (5.0)	109	
2025											
Jan	2,779	388 (14.0)	121 (31.2)	8 (6.6)		587	146 (24.9)	56 (38.4)	2 (3.5)	37	
Feb	2,032	118 (5.8)	27 (22.9)	2 (7.4)		300	36 (12.0)	3 (8.3)	1 (33.3)	13	
Mar	1,452	38 (2.6)	14 (36.8)	0		174	20 (11.5)	9 (45.0)	0	6	
Apr	1,257	26 (2.1)	8 (30.8)	0		132	2 (1.5)	2 (100)	0	6	
May	926	8 (0.9)	4 (50.0)	0		131	4 (3.1)	2 (50.0)	0	7	
Total	45,320	7,969 (17.6)	2,014 (25.3)	90 (4.5)		11,142	3,545 (31.8)	975 (27.5)	28 (2.9)		

*NA, not available. †Per 1,000 patients.

Among positive *M. pneumoniae* tests, we successfully sequenced 2,014 (25.0%) samples (Appendix Table 2). Resistance mutations occurred in 90 (4.5%) samples; 84 were A2063G, 5 were A2064G, and 1 was A2064T. Monthly resistance rates increased over time, peaking in October (8.7%) (Table; Figure). We detected no resistance mutations among 26 (36.1%) samples collected during March–May 2025. The resistance rate of the entire outbreak was higher than we previously reported (4.5% vs. 2.4%; p = 0.0152) (*3*). The median age of the 90 patients with MRMp was 9.1 years (IQR 6.3–12.3 years); 57 (63.3%) were male and 33 (36.7%) female (Appendix Table 1). Among 88 patients whose charts we reviewed, 27 (36.7%) had previous azithromycin exposure. Eighteen (20.0%) patients were hospitalized, and 4 (4.4%) received ICU care. Hospitalization, ICU admission, and co-detection rates were higher in patients with MRMp, although only hospitalization rates were significantly different between the 2 groups (Appendix Table 2).

Azithromycin prescribing rates from urgent care centers rose alongside MRMp rates and *M. pneumoniae*–positive rates (Table; Figure). As *M. pneumoniae* activity declined, azithromycin prescribing also decreased. Although azithromycin data were not available for all patients, testing at urgent care centers constituted 24.6% of *M. pneumoniae* testing and 44.5% of positive results for the whole study period (Table).

**Figure:**
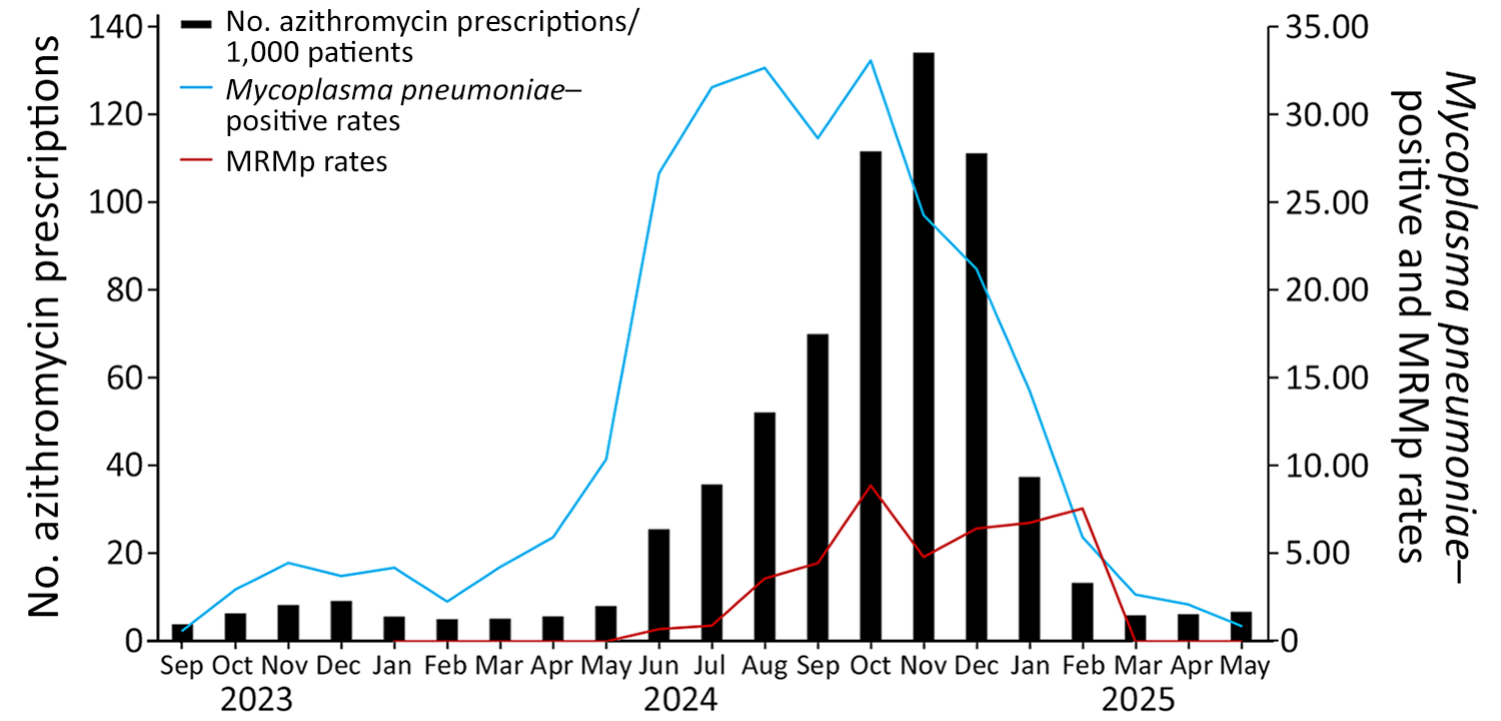
Monthly azithromycin prescriptions per 1,000 patients at Nationwide Children’s Hospital urgent care centers, *Mycoplasma pneumoniae* positive rates, and MRMp rates, Ohio, USA, September 2023–May 2025. Macrolide resistance was determined in a subset of samples. MRMp rates were not available during September 2023–December 2023. MRMp, macrolide-resistant *M. pneumoniae*.

This study revealed a substantial *M. pneumoniae* infection prevalence in central Ohio as the COVID-19 pandemic waned. More than 7,000 pediatric cases were identified in 21 months. Our findings confirmed a rising MRMp rate during the outbreak, peaking in October 2024 and returning to baseline after *M. pneumoniae* activity subsided. No MRMp was detected after March 2025. Prior macrolide exposure is a known risk factor for macrolide resistance (*4*), and rapid emergence of resistance during treatment has been reported (*5*,*6*). In this study, the rate of macrolide resistance correlated with changes in macrolides use, represented by azithromycin prescribing data (Figure). We postulate that increasing macrolide use in the community contributed to the emergence of MRMp, unlike MRMp epidemics in eastern Asia, where resistance was primarily driven by spread of resistant strains (*7*–*9*). As macrolide prescribing declined, the MRMp rate subsequently returned to baseline levels. Future research will include molecular characterization of the *M. pneumoniae* strains in this outbreak through genomic sequencing to support or refute our hypothesis.

The clinical effect of MRMp remains inconclusive. Prepandemic data from our center showed that MRMp was associated with longer hospital stays and increased ICU admissions. Conversely, a national surveillance study in the United States showed no significant difference (*10*). In our study, MRMp was linked to increased hospitalization. Those discrepancies might reflect variations in testing algorithms and study design. Further in-depth clinical characterization is needed to understand the underlying factors contributing to these outcomes.

Our study revealed a substantial *M. pneumoniae* infection prevalence in our patient population, confirmed increasing macrolide resistance, and showed that the MRMp rates fluctuated, possibly in correlation with azithromycin prescribing rates. Our findings highlight the importance of vigilant, real-time surveillance for MRMp to provide accurate information for management of children with *M. pneumoniae* infection.

Appendix Additional information on macrolide-resistant *Mycoplasma pneumoniae* in children, Ohio, USA.
