# Supplementary material for: Macrolide-Resistant Mycoplasma pneumoniae in Children, Ohio, USA
Source: Emerg Infect Dis. 2025 Dec;31(12):2341–3. doi: 10.3201/eid3112.251008 (PMC12782270; doi:10.3201/eid3112.251008)
Supplement: Appendix — Additional information on macrolide-resistant Mycoplasma pneumoniae in children, Ohio, USA. [file 25-1008-Techapp-s1.pdf]

Article DOI: https://doi.org/10.3201/eid3112.250118

EID cannot ensure accessibility for Supplemental Materials supplied by authors. Readers who have difficulty accessing supplementary content should contact the authors for assistance.

Macrolide-Resistant *Mycoplasma pneumoniae* in Children, Ohio, USA

Appendix

Appendix Table 1. Demographics and laboratory characteristics of study population by age groups

Characteristic	Age groups, y					p value*
	Total	<2	2–<6	6–<10	>10	
Tested	45,320	11,033	12,404	9,098	12,785	N/A
No. of MPN Positives (%)	7,969 (17.6)	375 (3.4)	1,763 (14.2)	2,624 (28.8)	3,207 (25.1)	<0.0001
Sequenced (%)	2,014 (25.3)	78 (20.8)	424 (24.1)	684 (26.1)	828 (25.8)	NS
Resistance (%)	90 (4.5)	1 (1.28)	20 (4.7)	29 (4.2)	40 (4.8)	NS
No. Positive Unique patients	7,649	344	1660	2,514	3,131	N/A
Gender M (%)	4,037 (52.8)	179 (52.0)	899 (54.2)	1,323 (52.6)	1,636 (52.3)	NS
No. of Medical encounters	7,741	350	1,680	2535	3,176	N/A
Inpatient (%)	657 (8.5)	67 (19.1)	188 (11.2)	155 (6.1)	247 (7.8)	<0.0001
ICU admission (%)	153 (2.0)	19 (5.4)	44 (2.6)	41 (1.6)	49 (1.5)	<0.0001
Outpatient (%)	7,084 (91.5)	283 (80.9)	1,492 (88.2)	23,80 (93.9)	2,929 (92.2)	<0.0001
UC (%)	3,545 (48.8)	121 (42.8)	726 (48.7)	1,251 (52.7)	1,447 (49.4)	0.0034
RP2.1 tested	795	104	196	210	285	N/A
Co-detection (%)	340 (42.8)	78 (75.0)	114 (58.2)	77 (36.7)	71 (24.9)	<0.0001

* Comparison was analyzed by Chi-squared. A p≤0.05 indicates that the groups are significantly different. ICU: intensive care unit; N/A: not applicable; NS: not significantly different; UC: urgent care centers.

Appendix Table 2. Demographics and laboratory characteristics in children tested for macrolide resistance

Characteristic	Total	No mutation detected	Resistant strain	p value*
No. sequenced	2,014	1,924	90	Not applicable
Median age, y (IQR)	9.0 (6.0, 11.9)	9.0 (6.0, 11.8)	9.1 (6.3, 12.3)	NS
Sex				
Male	1,105 (54.9)	1,048 (54.5)	57 (63.3)	NS
Inpatient (%)	245 (12.2)	227 (11.8)	18 (20.0)	0.02
Intensive care unit admission (%)	53 (2.6)	49 (2.6)	4 (4.4)	NS
Outpatient (%)	1,769 (87.8)	1,697 (88.2)	72 (80.0)	0.02
Urgent care centers (%)	975 (55.1)	947 (55.8)	28 (38.9)	NS
RP2.1 tested	176	158	18	N/A
Co-detection (%)	63 (35.8)	55 (34.8)	8 (44.4)	NS

* Comparison was analyzed by Chi-squared. A p≤0.05 indicates that the groups are significantly different. IQR: Interquartile range; N/A: not applicable; NS: not significantly different.

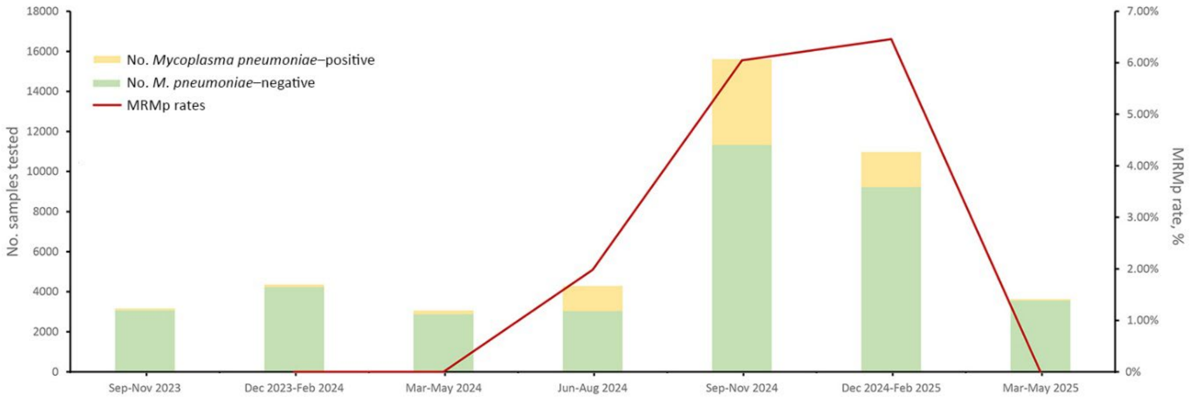

Appendix Figure. Quarterly testing volumes and rates of MRMp infections among children after the COVID-19 pandemic, Ohio, USA. Samples were tested for *Mycoplasma pneumoniae* infection during September 2023–May 2025. Macrolide resistance was determined in a subset of samples. MRMp rates were not available during September 2023–December 2023. MRMp, macrolide-resistant *Mycoplasma pneumoniae*.
